# Multiparametric cardiovascular magnetic resonance evaluation of myocardial perfusion, oxygenation, and energetics in hypertrophic cardiomyopathy following cardiac myosin inhibitor therapy

**DOI:** 10.1093/ehjci/jeae297

**Published:** 2024-11-28

**Authors:** Lucy E M Finnigan, Niklas Beyhoff, Zakariye Ashkir, Hugh Watkins, Stefan Neubauer, Betty Raman

**Affiliations:** Oxford Centre for Clinical Magnetic Resonance Research, Radcliffe Department of Medicine, Division of Cardiovascular Medicine, University of Oxford, John Radcliffe Hospital, Headley Way, Oxford OX3 9DU, UK; Oxford Centre for Clinical Magnetic Resonance Research, Radcliffe Department of Medicine, Division of Cardiovascular Medicine, University of Oxford, John Radcliffe Hospital, Headley Way, Oxford OX3 9DU, UK; Oxford Centre for Clinical Magnetic Resonance Research, Radcliffe Department of Medicine, Division of Cardiovascular Medicine, University of Oxford, John Radcliffe Hospital, Headley Way, Oxford OX3 9DU, UK; Oxford Centre for Clinical Magnetic Resonance Research, Radcliffe Department of Medicine, Division of Cardiovascular Medicine, University of Oxford, John Radcliffe Hospital, Headley Way, Oxford OX3 9DU, UK; Oxford Centre for Clinical Magnetic Resonance Research, Radcliffe Department of Medicine, Division of Cardiovascular Medicine, University of Oxford, John Radcliffe Hospital, Headley Way, Oxford OX3 9DU, UK; Oxford Centre for Clinical Magnetic Resonance Research, Radcliffe Department of Medicine, Division of Cardiovascular Medicine, University of Oxford, John Radcliffe Hospital, Headley Way, Oxford OX3 9DU, UK

A 60-year-old male with symptomatic obstructive hypertrophic cardiomyopathy underwent a transthoracic echocardiogram, multiparametric cardiovascular magnetic resonance (CMR) imaging (with adenosine stress), and ECG as part of an open-label observational trial of Aficamten (see Supplementary data online, *Figure S1*). At baseline, before initiation of treatment, echocardiography showed a resting left ventricular (LV) outflow tract gradient of 18 mmHg (162 mmHg post-Valsalva). CMR revealed basal septal hypertrophy of 27 mm in diameter (*Panel A*) with a small LV cavity size [indexed LV end diastolic volume (LVEDVi) 70 mL/m^2^] and high normal LV ejection fraction (LVEF) of 71%. Diffusion tensor CMR revealed abnormal fractional anisotropy (*Panel B*) and mean diffusivity (*Panel C*). Stress myocardial blood flow (MBF) and perfusion reserve (MPR) were impaired (0.99 mL/min/g and 1.68, respectively; *Panel D*); myocardial oxygenation response measured through blood oxygen level dependent (BOLD) upon vasodilatory stress was blunted (5%; *Panels E* and *F*). ^31^P CMR spectroscopy indicated impaired myocardial energetics (phosphocreatine/ATP ratio 1.68; *Panel M(i)*). ECG demonstrated ST abnormalities in the anterolateral leads (*Panel N* and Supplementary data online, *Figure S2*).

Twelve weeks of myosin modulator therapy resulted in improvements in symptoms and LV outflow tract gradient (rest: 13 mmHg, post-Valsalva: 61 mmHg). LVEDVi increased to 83 mL/m^2^, and LVEF decreased to 55% (*Panel G*). There was an increase in fractional anisotropy (*Panel H*) and reduction in mean diffusivity (*Panel I*). Stress MBF and MPR increased (*Panel J*), alongside stress oxygenation (15%, *Panels K* and *L*). The phosphocreatine/ATP ratio increased to 1.90 following treatment (*Panel M(ii)*). ECG revealed improvements in repolarization abnormalities (*Panel O*). Non-invasive markers of myocardial fibrosis remained unaffected (Supplementary data online, *Figure S3*).

This study demonstrates how multiparametric CMR can be used to evaluate the effects of novel drugs, such as cardiac myosin inhibitors, on myocardial perfusion, oxygenation, and energetics.

**Figure:**
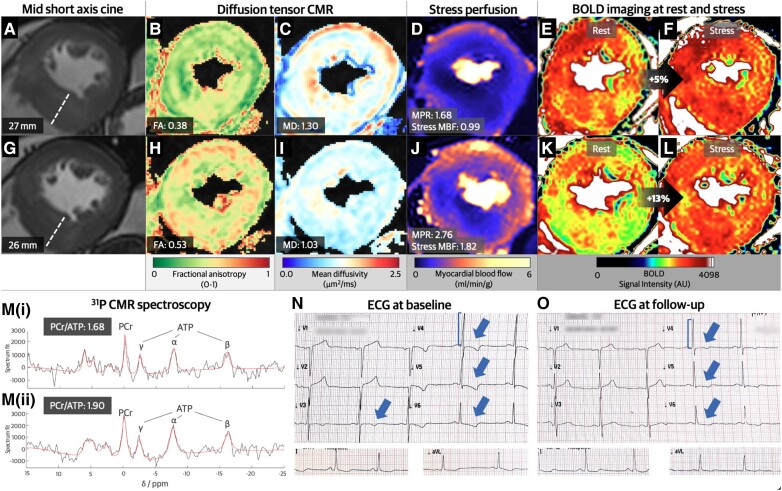


Supplementary data are available at *European Heart Journal - Cardiovascular Imaging* online.

**Funding:** Scans were funded by the National Institute for Health and Care Research (NIHR) Oxford Biomedical Research Centre. L.E.M.F. is supported by the NIHR Oxford Biomedical Research Centre and the British Heart Foundation Oxford Centre of Research Excellence. Z.A. is a recipient of a British Heart Foundation Clinical Research Training Fellowship (FS/CRTF/21/24144). B.R. is funded by a Wellcome Career Development Award fellowship (302210/Z/23/Z).

**Data availability:** The data underlying this article will be shared on reasonable request to the corresponding author.

## Supplementary Material

jeae297_Supplementary_Data

