# Supplementary material for: Multiparametric cardiovascular magnetic resonance evaluation of myocardial perfusion, oxygenation, and energetics in hypertrophic cardiomyopathy following cardiac myosin inhibitor therapy
Source: Eur Heart J Cardiovasc Imaging. 2024 Nov 28;26(2):378. doi: 10.1093/ehjci/jeae297 (PMC11781825; doi:10.1093/ehjci/jeae297)
Supplement: jeae297_Supplementary_Data [file jeae297_supplementary_data.pdf]

# - SUPPLEMENTARY MATERIAL –

Multiparametric cardiovascular magnetic resonance evaluation of myocardial perfusion, oxygenation, and energetics in hypertrophic cardiomyopathy following cardiac myosin inhibitor therapy

Lucy E.M. Finnigan[1†], Niklas Beyhoff[1†], Zakariye Ashkir[1], Hugh Watkins[1], Stefan Neubauer[1], Betty Raman[1]

[1] Oxford Centre for Clinical Magnetic Resonance Research, Radcliffe Department of Medicine, Division of Cardiovascular Medicine, University of Oxford, Oxford, United Kingdom [†] These authors contributed equally.

Supplementary methods

The FOREST-HCM trial (NCT04848506) received site-specific institutional ethics approval and was funded by Cytokinetics. The National Research Ethics Committee approved the additional CMR imaging study protocol (REC reference: 12/LO/1979), and written informed consent was obtained from the patient.

Study protocol

FOREST-HCM is an ongoing open-label extension study assessing the efficacy and safety of a myosin modulator (Aficamten) in patients with hypertrophic cardiomyopathy (HCM). In accordance with the inclusion criteria of FOREST-HCM, the patient had previously participated in a phase three randomised controlled trial of Aficamten (SEQUOIA-HCM)[1]. There was a three-month period following the cessation of treatment during SEQUOIA-HCM and the start of treatment in FOREST-HCM (**Figure S1**). The patient attended a study visit before initiation of Aficamten (baseline visit) and a follow-up visit after 12 weeks of treatment. Initial dose of Aficamten was 5 mg which was then titrated to 20 mg following regular echocardiography examinations. During the two study visits, the patient underwent physical

assessment, a 12-lead ECG, echocardiography, and additional comprehensive cardiac magnetic resonance (CMR) imaging.

Echocardiography

A standard transthoracic echocardiogram was performed to measure left ventricular outflow tract gradient at rest and during Valsalva manoeuvre.

CMR protocol

All CMR imaging was performed on a clinical 3T scanner (Siemens MAGNETOM Prisma, Germany) at the Oxford Centre for Clinical Magnetic Resonance Research, University of Oxford, United Kingdom. Cine images were acquired using standard methods. Left ventricular volumes, systolic function, wall thickness, and mass were determined using cvi42 (Circle Cardiovascular Imaging, Canada). Native T1 was acquired at the mid-ventricular short-axis slice using the Shortened MOdified Look-Locker Inversion recovery sequence (ShMOLLI). Fractional anisotropy and mean diffusivity were assessed at mid-myocardial level during diastolic pause using an ECG-gated Stimulated Echo Acquisition Mode (STEAM) single-shot echo planar imaging sequence with monopolar diffusion encoding, as previously described[2]. Diffusion sequence parameters were fat saturation, TR = 2 RR intervals, TE = 22 ms, BW = 2442 Hz/pixel, GRAPPA parallel imaging acceleration factor of 2, FOV = 360 × 135 mm, acquisition matrix = 128 × 48 pixels, spatial resolution = 2.8 × 2.8 × 8 mm^3, interpolated to 1.4 × 1.4 × 8 mm^3. Diffusion tensor CMR post-processing was performed using custom-built software using MATLAB (MathWorks, United States)[2].

Myocardial oxygenation was evaluated by FLASH normalised T2-prepared steady-state free precession Blood Oxygen Level-Dependent (BOLD) imaging, as previously described[3]. The

mean BOLD signal intensity was assessed at rest and under vasodilator stress (adenosine 140 micrograms/kg/min) in a mid-ventricular short-axis slice. Vasodilator stress myocardial oxygenation was defined as the relative change in BOLD signal intensity from rest to stress (ΔBOLD%). Similarly, mid-myocardial short-axis perfusion images were acquired at rest and during adenosine stress[4]. Perfusion imaging was performed over 60 heartbeats after administering a bolus of 0.05 mmol/kg gadolinium-based contrast agent (Dotarem, Guerbet, France). Perfusion maps were reconstructed automatically, and the average myocardial blood flow was assessed. Myocardial perfusion reserve (MPF) was defined as the ratio between myocardial blood flow during vasodilator stress and at rest.

The ratio of phosphocreatine to average ATP (PCr/ATP), a marker of overall myocardial energetics, was assessed via ^{31}P magnetic resonance spectroscopy as described before[5]. Depth-resolved surface coil spectroscopy and data postprocessing were carried out according to a standard operating protocol.

Acknowledgement

We thank the physics and radiographer teams at OCMR for the expert support of this study (special thanks to Drs Elizabeth M Tunnicliffe, Ladislav Valkovič, Peter Gatehouse and Ms Miriam Lacherie), and Dr Rina Ariga for the insightful discussions on diffusion tensor CMR.

Supplementary references

1. Maron MS, Masri A, Nassif ME, Barriales-Villa R, Arad M, Cardim N, Choudhury L, Claggett B, Coats CJ, Düngen H-D, Garcia-Pavia P, Hagège AA, Januzzi JL, Lee MMY, Lewis GD, Ma C-S, Michels M, Olivotto I, Oreziak A, Owens AT, Spertus JA, Solomon SD, Tfelt-Hansen J, Van Sinttruije M, Veselka J, Watkins H, Jacoby DL, Heitner SB, Kupfer S, Malik FI, Meng L, Wohltman A, Abraham TP. Aficamten for Symptomatic Obstructive Hypertrophic Cardiomyopathy. *N Engl J Med* 2024;**390**:1849–1861.

2. Ariga R, Tunnicliffe EM, Manohar SG, Mahmod M, Raman B, Piechnik SK, Francis JM, Robson MD, Neubauer S, Watkins H. Identification of Myocardial Disarray in Patients With Hypertrophic Cardiomyopathy and Ventricular Arrhythmias. *J Am Coll Cardiol* 2019;**73**:2493–2502.

3. Raman B, Tunnicliffe EM, Chan K, Ariga R, Hundertmark M, Ohuma EO, Sivalokanathan S, Tan YJG, Mahmod M, Hess AT, Karamitsos TD, Selvanayagam J, Jerosch-Herold M, Watkins H, Neubauer S. Association Between Sarcomeric Variants in Hypertrophic Cardiomyopathy and Myocardial Oxygenation: Insights From a Novel Oxygen-Sensitive Cardiovascular Magnetic Resonance Approach. *Circulation* 2021;**144**:1656–1658.

4. Kotecha T, Martinez-Naharro A, Boldrini M, Knight D, Hawkins P, Kalra S, Patel D, Coghlan G, Moon J, Plein S, Lockie T, Rakhit R, Patel N, Xue H, Kellman P, Fontana M. Automated Pixel-Wise Quantitative Myocardial Perfusion Mapping by CMR to Detect Obstructive Coronary Artery Disease and Coronary Microvascular Dysfunction. *JACC Cardiovasc Imaging* 2019;**12**:1958–1969.

5. Monga S, Valkovič L, Myerson SG, Neubauer S, Mahmod M, Rider OJ. Role of Cardiac Energetics in Aortic Stenosis Disease Progression: Identifying the High-risk Metabolic Phenotype. *Circ Cardiovasc Imaging* 2023;**16**.

Supplementary figures

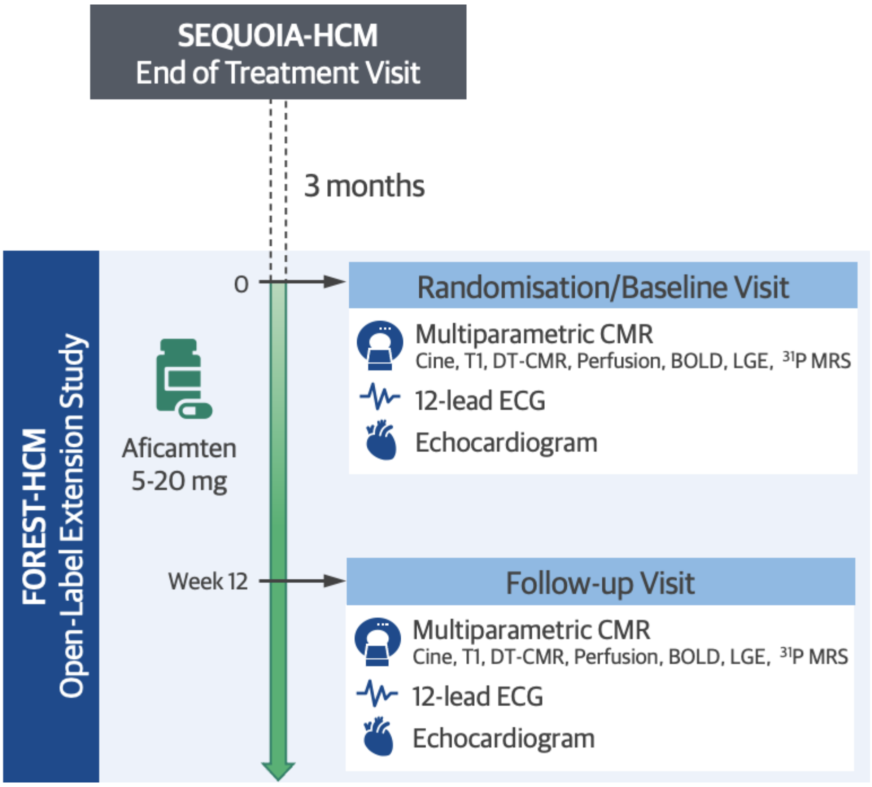

Figure S1: Overview about study protocols.

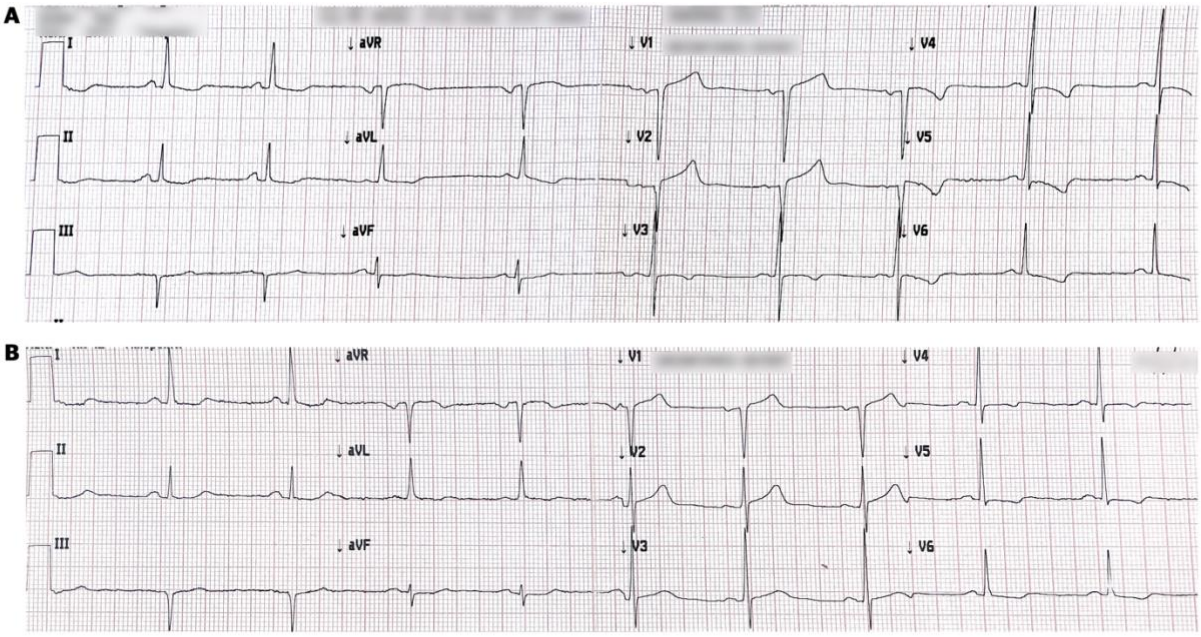

Figure S2: ECG recording (**A**) before and (**B**) after 12 weeks of treatment with Aficamten.

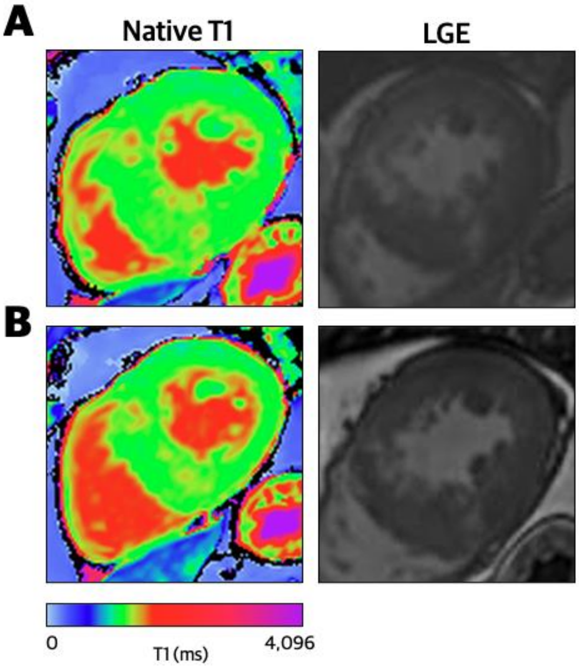

Figure S3: Native myocardial T1 mapping and late gadolinium enhancement (LGE) imaging of the LV mid slice at (**A**) baseline and (**B**) after 12 weeks of Aficamten. Native myocardial T1 times remain unchanged after 12 weeks of Aficamten (baseline: 1203 ± 77 ms; at 12 weeks: 1199 ± 67 ms). Similarly, cardiac myosin inhibitor therapy did not modify the overall fibrotic burden as assessed by late gadolinium imaging.
